# Two New Species of the Genus *Caryanda* Stål, 1878 (Orthoptera: Acrididae) from Yunnan, China Identified Based on Morphological and Molecular Data †

**DOI:** 10.3390/insects17020164

**Published:** 2026-02-02

**Authors:** Fangting Li, Hong Song, Yongmei Zhao, Jianyu Chen, Jinchen Yang, Miao Li, Benyong Mao

**Affiliations:** 1 College of Agriculture and Biological Science, Dali University, Dali 671003, China; lfangting2025@163.com (F.L.); fslaaaaa@163.com (H.S.); 2 Nangunhe National Nature Reserve Management and Protection Bureau, Cangyuan 677400, China; 15906933013@163.com; 3 Key Laboratory of Zoological Systematics and Application, School of Life Sciences, Institute of Life Sciences and Green Development, Hebei University, Baoding 071002, China; chenjianyu0618@163.com; 4 Independent Researcher, Luoyang 471000, China; 18317518102@163.com

**Keywords:** grasshoppers, Acridoidea, *Caryanda*, mitochondrial genome, integrative taxonomy, new species

## Abstract

*Caryanda* is a group of morphologically diverse, non-flying locusts. In November 2024, we first discovered two unknown species of the genus in Nangonghe Nature Reserve, Yunnan Province. In this study, the effectiveness of these two new species was verified by integrating morphological, morphometric, and mitochondrial genomic methods. Finally, two new species, *Caryanda gengmaensis* Mao et Li sp. nov. and *Caryanda analbomaculata* Mao et Li sp. nov., were proposed and described in detail, and two similar species color plates were attached: *C. cyanonota* Mao & Li, 2015 and *C. albomaculata* Mao, Ren & Ou, 2007.

## Simple Summary

Species of *Caryanda* are small-sized, flightless grasshoppers with residual wings, commonly found in the semi-shaded, moist grasses under broad-leaved forests in Yunnan. They primarily feed on Gramineae plants, exhibit highly diverse coloration, and are narrowly distributed in isolated habitats. However, populations have seriously declined in recent years because of the substitution of original vegetation and the extensive use of pesticide and are now restricted to only certain areas. This population decline has disrupted the original ecological network and may even lead to species extinction. Since the late 1990s, our team has collected locust samples nearly a hundred times in various counties and villages in Yunnan Province, trying to understand the diversity and biology of this group. Based on morphology, morphometry, and mitochondrial genome data, two new species of *Caryanda* collected from Gengma County, Yunnan Province were described. The mitochondrial genome information given by this study provides an important and valuable reference for the identification of *Caryanda* species in the future.

## 1. Introduction

Insects are the most diverse group of animals on the planet [1]. To catalog the vast number of species, naturalists have classified and described living beings mainly based on morphological features for centuries. To date, over one million species have been described, while millions remain undescribed or undiscovered. However, in traditional taxonomy, the overestimation of the classification value of some morphological features easily leads to paraphyly and taxonomic inflation, while convergent characters can lead to polyphyly [2,3,4,5]. Due to the limitations of morphology, molecular approaches can overcome the challenges associated with morphological species identification and delimitation of cryptic species, leading to higher taxonomic resolution in ecosystem assessments [6,7]. For example, insect’s mitochondrial genome having the advantages of maternal inheritance, simple structure, compact gene arrangement, and high stability [8,9,10], has proven effective in biodiversity assessments and taxonomic revisions [11,12,13,14,15]. In recent years, molecular data has been more and more widely used in the discovery and description of new species or cryptic species, molecular identification of species, and phylogenetic studies [16,17,18,19,20,21]. However, molecular data alone cannot be used as an absolute standard for species definition [22,23,24]. Nowadays, as the cornerstone of exploring biodiversity, insect taxonomy is far more than naming and cataloging species. In the context of today’s accelerating global change and biodiversity loss, taxonomy has evolved from a traditional morphological description and systematic combing to a comprehensive discipline that integrates multi-dimensional evidence and responds to urgent ecological challenges [25,26,27,28].

The genus *Caryanda* Stål, 1878 (Acrididae) possesses a large number of species, which are ecologically and economically important both as a food source of predators and as agricultural pests in Yunnan. So far, 89 species of the genus are known worldwide [29], mainly in China (72 species, including 29 species in Yunnan), the Indo-China Peninsula (5 species, including 3 species in Vietnam, and 2 species in Thailand), and the Indian subcontinent (8 species, including 6 species in India, 1 species in Nepal, and 1 species in Bhutan), with a small number of species in the Malay Archipelago (1 species), Central Africa (1 species), and West Africa (2 species). Previous studies proposing new species in this genus have primarily relied on morphological differences, such as body coloration, the shape of male’s epiproct and cerci, genitalia, and the shape of female’s subgenital plate. In addition, some species were described only on the basis of a single sex (e.g., *C. methiola* Chang, 1939, *C. gyirongensis* Huang, 1981, *C. albufurcula* Zheng, 1988, *C. gulinensis* Zheng, Shi & Chen, 1994, and *C. triodontoides* Zheng & Xi, 2008, etc.) or a single type (e.g., *C. gyirongensis* Huang, 1981 and *C. triodontoides* Zheng & Xi, 2008, etc.) in the early research, and either the range of feature variation or genital features were not provided, which potentially raise questions regarding species validity. Therefore, alongside morphological differences, integrating molecular methods and other methodologies proves more effective in confirming species validity and authenticity.

Since the late 1990s, our team has collected locust samples nearly a hundred times in Yunnan Province, describing 20 new species of the genus, trying to understand their diversity and biology. Recently, we discovered two unknown species collected in Gengma County, Yunnan Province, which showed similarities to two known species, respectively, in morphology and colouration, but with a higher inhabiting altitude and a delayed eclosion peak. The purpose of this study is to compare the differences between unknown species and morphologically similar species by using a comprehensive taxonomic method combining morphology and mitochondrial genome data, and to explore their phylogenetic relationships. If it is confirmed that there are sufficient morphological and molecular differences, the new species will be described further.

## 2. Materials and Methods

### 2.1. Origin of the Specimens

In November 2024, we first discovered two colorful, previously unknown *Caryanda* species within the Nangun River Nature Reserve in Yunnan, where adult populations were extremely limited. During a follow-up survey in January 2025, we successfully collected 30 valuable specimens. This study is based on specimens preserved in the Collection of the Biological Science Museum, Dali University, China [BMDU] and collected from Gengma County, Yunnan Province, China.

### 2.2. Morphological Study

The morphological terminology and measurements followed those of Uvarov (1966) [30], Dirsh (1975) [31], and Ingrisch et al. (2004) [32]. The terminology of male genitalia followed that of Dirsh (1956) [33]. The color Figure 3, Figure 4, Figure 5, Figure 7, Figure 8 and Figure 9 were prepared using a digital microscope (Keyence VHX-7000N, Keyence Corporation, Osaka, Japan), and Figure 6 and Figure 10, were photographed by a Canon digital camera (EOS 60D) or smartphone (Xiaomi 14). The final illustrations were prepared into plates with Adobe Photoshop^®^ CS2 software (Adobe Photoshop 2023). The type specimens were deposited in the Biological Science Museum, Dali University, China [BMDU].

The measurements generally used for grasshoppers are defined as below:

BL: body length, the length from the apex of fastigium to the apex of subgenital plate;

PNL: pronotum length, the length from the anterior margin of pronotum to the posterior margin;

HFL: hind femur length, the maximum distance from the base of hind femur to the apex;

HFW: hind femur width, the maximum distance from the base of hind femur midian keel of upper side of hind femur to median keel of lower side.

EL: epiproct length, from the tenth abdominal tergite widely divided in middle to epiproct apex;

EW: epiproct width, the maximum distance on both sides of epiproct;

CL: cerci length, the maximum distance from the base of cerci to the apex;

CW: cerci width, the maximum distance from the base of cerci;

FSPL: Female subgenital plate length;

FSPW: Female subgenital plate width.

The quantitative data were measured under a stereomicroscope using an ocular micrometer. The two-sample Wilcoxon test (also known as “Mann–Whitney” test) were performed using the function “wilcox.test()” in the “stats” package of R 4.4.0.

### 2.3. DNA Extraction, Amplification, and Sequencing

Eleven specimens of five Chinese *Caryanda* species were sequenced in this study, and two sequences of the genus *Caryanda* were downloaded directly from GenBank. In addition, we downloaded NCBI data of *Alulatettix yunnanensis* as the outgroup (Table 1).

The sample sequencing work was completed by Shanghai Origingene Biotechnology Co., Ltd. (Shanghai, China). In this study, CTAB method [34] was used to extract the muscle DNA from the hind femur of the specimen, and the purity and integrity of the sample DNA were detected and analyzed by agarose gel electrophoresis and spectrophotometry. All sequencing processes were performed by Shanghai Origingene Biotechnology Co., Ltd. (Shanghai, China). The Illumina HiseqXten platform was used for sequencing, and the total length of the original data was about 50 × 800 bp. The quality control and pruning of the original reads were completed by CLC Genomics Workbench v7.0.4 software, and the parameters were set as the default values.

### 2.4. Data Analysis

After obtaining clean reads, they were assembled using NOVOPlasty-master v4.2.1 software and *Caryanda neoelegans* (GenBank accession number NC 036750) was used as the reference genome. Multiple sequence alignments of 13 PCGs, 22 tRNAs, and 2 rRNAs were performed using MAFFT in PhyloSuite v1.2.3 software, of which 13 PCGs were optimized using MACSE (implemented in PhyloSuite v1.2.3) [35]. The nucleotide sequences were aligned using Gblocks v0.91b to remove the ambiguous sites [36], while the two rRNAs and 22 tRNAs sequences were aligned using the Q-INS-i algorithm that takes the secondary structure into account in MAFFT (implemented in PhyloSuite v1.2.3) software. Phylogenetic trees were constructed using the maximum likelihood (ML) method through PhyloSuite v1.2.3 multi-gene joint analysis software [37]. In addition, intergroup genetic distances were calculated for seven species of *Caryanda* using the Kimura 2-parameter (K2P) model in MEGA 11.

## 3. Results

### 3.1. Phylogeny Analysis of 7 Caryanda Species Based on Mitochondrial Genome Sequences

A total of 13 mitochondrial genome sequences of 7 *Caryanda* species were used for phylogenetic analysis, of which 11 sequences of five Chinese *Caryanda* species were first sequenced in this study and the other 2 sequences were downloaded from the GenBank.

The newly sequenced mitochondrial genome of five species were assembled and annotated, length of 15,532 bp (*C. gengmaensis* Mao et Li sp. nov.), 15,454–15,455 bp (*C. analbomaculata* Mao et Li sp. nov.), 15,452–15,750 bp (*C. cyanonota*), 15,451–15,452 bp (*C. albomaculata*), and 15,435–15,439 bp (*C. azurea colourfula*). These mitochondrial genomes are typical double-stranded closed-loop molecular structures, contain 37 gene fragments: 13 protein-coding genes (PCGs), 22 transfer ribonucleic acids (tRNA), 2 ribosomal ribonucleic acids (rRNA), and 1 control region fragment (non-coding region, D-loop).

**Figure 1:**
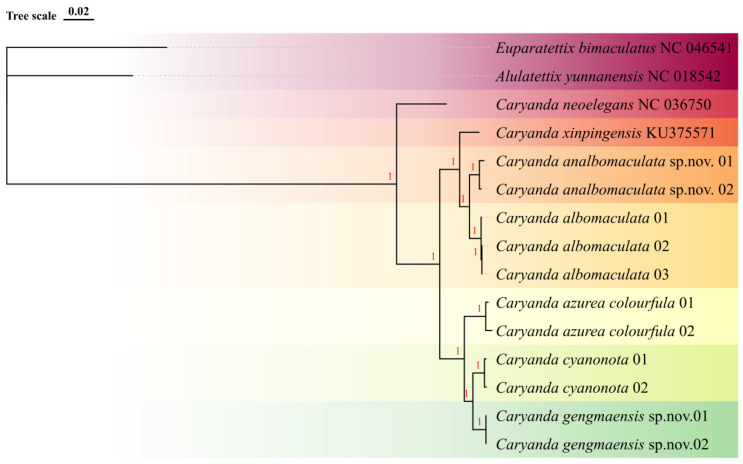
Bootstrap consensus tree generated with the Bayesian (BI) method, showing the genetic relationship between isolated *Caryanda* species obtained by mitochondrial genome sequences. Each species is highlighted with a different color. The numbers on the nodes represent Bayesian posterior probabilities.

With *Alulatettix yunnanensis* (NC 018542) and *Euparatettix bimaculatus* (NC 046541.1) as outer group, the consensus trees generated by maximum likelihood method and Bayesian method based on mitochondrial genome sequences have the same topological structure, revealing the consistent phylogenetic relationship. The phylogenetic tree showed that all *Caryanda* species clustered independently in the BI tree, forming seven distinct branches with strong bootstrap support (100%), without any irregularities, such as alternations or anomalies (Figure 1). The phylogeny analysis revealed three major clades: clade Ⅰ containing *C. neoelegans*; clade Ⅱ containing *C. azurea colourfula*, *C. gengmaensis* Mao et Li sp. nov. and *C. cyanonota*; and clade Ⅲ containing *C. xinpingensis*, *C. analbomaculata* Mao et Li sp. nov., and *C. albomaculata*. Among them, *C. gengmaensis* Mao et Li sp. nov. and *C. cyanonota* are closely related as a sister group, while *C. analbomaculata* Mao et Li sp. nov. and *C. albomaculata* are closely related as another sister group. The results of phylogenetic studies showed that the monophyly of each species was supported, and were also consistent with the results of morphological studies (Figure 1).

**Figure 2:**
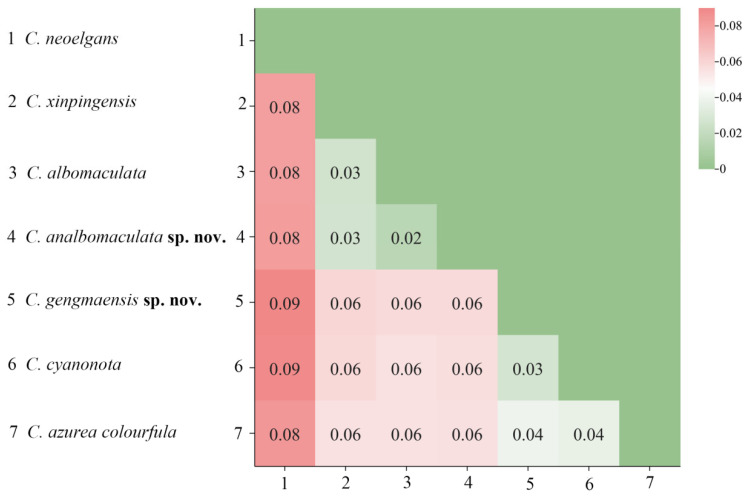
The genetic distance map between species based on the complete mitochondrial genome sequences.

Based on the Kimura 2-parameter (K2P) model, genetic distances among 7 species of *Caryanda* were calculated. The results showed that the genetic distance between *C. gengmaensis* Mao et Li sp. nov. and *C. cyanonota* was 0.03, while the genetic distance between *C. analbomaculata* Mao et Li sp. nov. and *C. albomaculata* was 0.02, a minimum value among them (Figure 2).

### 3.2. Quantitative Traits Analysis

Typically, the genetic divergence within species for mitochondrial genome sequences is between 0% and 2% [38]. In view of the only 2% interspecific genetic distance between *C. analbomaculata* Mao et Li sp. nov. and *C. albomaculata*, we tried to reveal the differences between them by quantitative traits to prove the authenticity of the new species. Therefore, a significance test of quantitative trait differences between the two was carried out.

We measured a total of 8 quantitative traits and 3 related ratios of male individuals and 6 quantitative traits and 2 related ratios of female individuals, and calculated 2 correlation indexes to reveal significance test of the difference in quantitative characters between the two species. The results show that there are 5 features (“male body length”, “pronotum length”, “hind femur length”, “cercus length”, and “cercus width”), and 2 correlation indexes (“ratio of male hind femur length to hind femur width” and “ratio of female subgenital plate length to subgenital plate width”) between the two species show significant differences (Table 2 and Table 3).

### 3.3. Morphological Taxonomy

**Genus *Caryanda* Stål, 1878**

*Caryanda* Stål, 1878: 47 [39]. Type species: *Acridium* (*Oxya*) *spurium* Stål, 1860 [39].

*Dibastica* Giglio-Tos, 1907: 9 [40]; Hollis, 1975: 217 (junior synonym of *Caryanda*) [41].

*Austenia Ramme*, 1929: 331 (nomen preoccupatum, nec Nevill, 1878) [42]; Hollis, 1975: 217 (junior synonym of *Caryanda*) [41].

*Austeniella* Ramme, 1931: 934 (replacement name for *Austenia*) [43]; Hollis, 1975: 217 (junior synonym of *Caryanda*) [41].

*Tszacris* Tinkham, 1940: 313 [44]; Li, Xia, et al., 2006: 103 (junior synonym of *Caryanda*) [45].

*Sinocaryanda* Mao & Ren, 2007: 366 [46]; Mao, Ren & Ou, 2011: 60, 296 (junior synonym of *Caryanda*) [47].

**Generic diagnosis.** Body small sized. Pronotum with posterior margin incised in middle, median carina weakly indicated, lateral carina absent. Tegmina scale-like, laterally situated. Hind femora with lower genicular lobes spined. Hind tibiae cylindrical, not expanded in apical half, external apical spines present. In male, furculae horizontally present or rarely absent on hind margin of 10th abdominal tergite; subgenital plate moderately conical.

We have found two unknown grasshopper species, which completely match the generic diagnosis well, resembling two known species *Caryanda cyanonota* Mao & Li, 2015 and *Caryanda albomaculata* Mao, Ren & Ou, 2007, respectively. We verified them by morphological and molecular methods and confirmed that they are effective species and are named *Caryanda gengmaensis* Mao et Li sp. nov. and *Caryanda analbomaculata* Mao et Li sp. nov.

#### 3.3.1. *Caryanda gengmaensis* Mao et Li sp. nov. (Figure 3, Figure 5 and Figure 6)

Chinese common name: 耿马卵翅蝗

**Type Material.** Holotype: male, CHINA: Gengma County, Yunnan Province, 23°38′ N, 99°23′ E, alt. 2066 m, 7 January 2025, leg. Fangting Li, Xun Wang and Honglei Yu. Paratypes: 10 males, 11 females, same data as holotype; 2 males, CHINA: Gengma County, Yunnan Province, 29 November 2024, leg. Jianyu Chen and Yongmei Zhao. All type specimens are deposited in BMDU.

**Diagnosis.** This new species is similar to *C. cyanonota* Mao & Li, 2015, but differs from the latter in: (1) the apex of male epiproct obtuse-angled against rectangular in *C. cyanonota*; (2) the apex of male cerci obtusely acute, in *C. cyanonota* the apex acute; (3) the lophi of epiphallus trapeziform, base narrower than top, whereas the lophi nearly oblong, base as wide as top in *C. cyanonota*; (4) the apical valves of penis S-shaped, apical part strongly broader than sub apical part in lateral view, whereas in the latter the apical valves of penis L-shaped, apical part slightly broader than sub apical part; (5) the light marks on body back being greenish yellow and varying in size instead of blue and even in size in *C. cyanonota*; (6) male hind femora almost entirely red against only 3/5 red in *C. cyanonota*; (7) the black transverse sulci on pronotum of male very thick, whereas the sulci obviously thin in the latter; and (8) female copper with metal color instead of light brown in *C. cyanonota*.

**Etymology.** The specific epithet ‘gengmaensis’ reflects the type locality.

**Description.** Body small, stouter in female.

**Figure 3:**
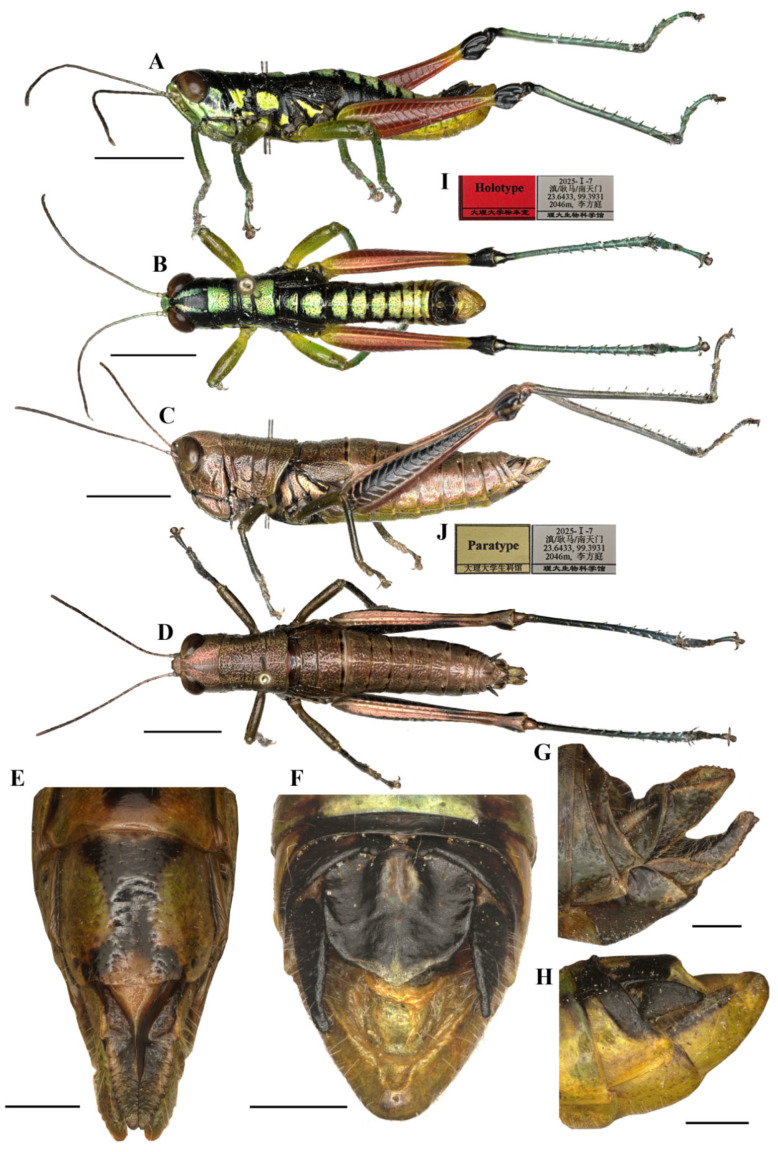
Type specimen picture of *Caryanda gengmaensis* Mao et Li sp. nov. (**A**,**B**) Male habitus, lateral and dorsal views; (**C**,**D**) female habitus, lateral and dorsal views; (**E**) female terminalia in ventral view; (**F**) male terminalia in dorsal view; (**G**,**H**) female and male terminaliae in lateral view; (**I**,**J**) holotype and paratype labels, respectively. Scale bars: 5 mm (**A**–**D**), 1 mm (**E**–**H**).

**Head** (Figure 3A–D). Head shorter than pronotum; fastigium broad, nearly flat in dorsal view, weakly prominent in lateral view, width in front of eyes about 1.60–2.20 (1.90, mean, n = 5, male) (the same below) or 2.00–2.50 (2.30, female) times larger than length, interocular distance 0.80–1.00 (0.95, male) or 1.10–1.20 (1.15, female) times width of frontal ridge between antennae. Face sloping; frontal ridge laterally straight with shallow-longitudinal sulcus, lateral margins nearly parallel except somewhat extended around median ocellus. Lateral facial keels thick and straight. Antennae filiform, retrad reaching (female) or surpassing (male) the base of hind femora (male), median 7–9 segments about 1.90–2.50 (2.30, male) or 1.80–2.20 (2.00, female) times as long as wide. Eyes oval, longitudinal diameter about 1.30–1.60 (1.37, male) or 1.50–1.70 (1.57, female) times as long as horizontal diameter, and about 2.38–2.56 (2.66, male) or 1.95–2.25 (2.06, female) times as long as subocular furrow.

**Thorax** (Figure 3A–D). Pronotum in both genders nearly cylindrical, surface foveolate, anterior margin nearly straight, posterior margin nearly straight and with a wide and shallow notch at the middle; median carina indistinct, lateral carinae absent; three transverse sulci distinct; prozona 1.70–2.65 (2.10, male) or 2.23–2.67 (2.30, female) times as long as metazona; lateral lobe with posterior margin distinctly concave, posteroventral corner nearly rectangular (male) or obtusely angular (female). Prosternal spine conical, straight, apex weakly acute (male, female). Mesosternal interspace about 1.33–1.50 (1.60, male) or 0.89–1.13 (0.98, female) times longer than minimum width; mesosteral lobes nearly square, 1.10–1.25 (1.20, male) or 1.20–1.65 (1.33, female) times wider than long. Metasternal lobes almost contiguous (male) or separated (female). Tegmina narrow squamiform, length 2.00–2.60 (2.20, male) or 2.00–2.30 (2.20, female) times larger than maximum width, reaching at or just surpassing beyond posterior margin of 1st abdominal tergite in both sexes. Hind femur with upper carina smooth, terminating in an acute angle; apex of lower knee lobes spinous. Hind tibia with apical half nearly cylindrical, with 7–8 external and 9–10 internal spines on dorsal side; external apical spine present. Abdomen with median keel. Tympana opening distinct, oval.

**Abdomen** (Figure 3E–H). Male genitalia (Figure 3F,H). Tenth abdominal tergite widely divided in middle, with small furculae (Figure 3F). Epiproct roundedly pentagonal, width at base somewhat larger than length; basal half with broad median longitudinal sulcus, lateral areas longitudinally elevated; lateral margin and hind margin forming a rounded obtuse angle; posterior margin triangular, apex obtuse-angled (Figure 3F). Cerci triangular, surpassing apex of epiproct, compressed laterally, gradually narrowing apically, apex obtusely sharp, faintly bended entad. Subgenital plate long conical, apex round (Figure 3H). Epiphallus with lophi near trapeziform, pointing dorsad, base narrower than top in posterior view, apical inner angle roundedly acute; ancorae fingerlike, apically blunt; bridge divided in middle (Figure 5A,B). Phallic complex (Figure 5D–F): apical valves of penis and cingular valves obviously extended out of hind margin of cingular rami; apical valves of penis compressed laterally, retrad upcurved and then upcurved, S-shaped, apical part strongly broader than sub apical part in lateral view; cingular valves upcurved in lateral view, fused apically.

Female genitalia (Figure 3E,G). Epiproct nearly triangular, with a transverse crease in the middle and a middle longitudinal sulcus in the basal half. Cerci conical, apex blunt, not reaching apex of epiproct (Figure 3G). Subgenital plate broad, near quadrate, median area concaved, posterior margin nearly straight but weakly excised near both sides. Valves of ovipositor with dentes along margins (Figure 3E).

**Coloration.** The following notes according to fresh specimens.

Male (Figure 6B) Body greenish yellow. Genae with oblique black strokes below eyes. Head with a triangular black mark on back. Antennae and eyes brown. Postocular bands black, continued to pronotum, tegmina and 8th abdominal tergite. The greenish yellow mark on the disk of pronotum divided into four small marks by three black transverse grooves, the second mark, which is sometimes not obvious, and the third sometimes dotted a pair of obvious small black spots. Pronotum with lateral lobes having two greenish yellow spots, inferior margins black. Meso- and meta-thoraxes black with two greenish yellow triangular marks on terga and pleura, respectively. Tegmina black. Fore and mid legs green. Hind femora dull red except the base slightly yellow and a yellow ring before black knee; hind tibiae blue. Abdominal terga black with eight greenish yellow maculations on back, but 9th–10th terga black; abdominal sterna and terminalia yellow. Cerci and Epiproct black. Subgenital plate greenish yellow. The color of dried specimens is similar to that of fresh ones, except 14 greenish yellow spots discoloring to dirty yellow on back of body.

**Figure 4:**
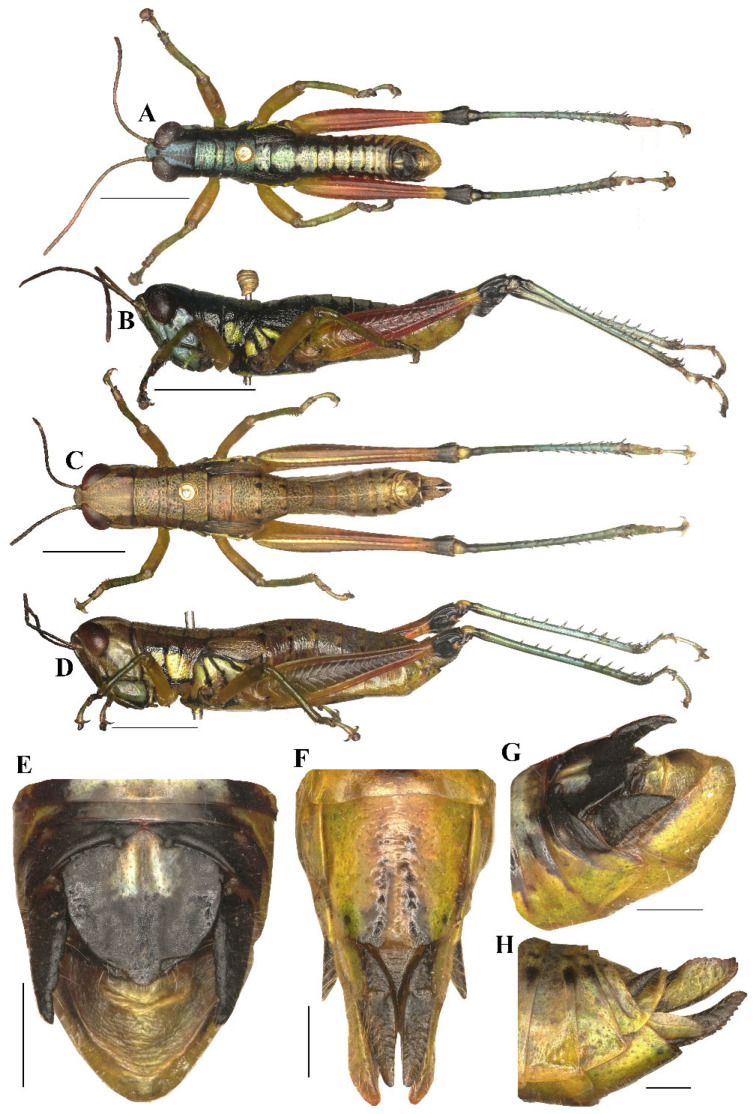
*Caryanda cyanonota* Mao & Li, 2015. (**A**,**B**) Male habitus, lateral and dorsal views; (**C**,**D**) female habitus, lateral and dorsal views; (**E**) female terminalia in ventral view; (**F**) male terminalia in dorsal view; (**G**,**H**) female and male terminaliae in lateral view. Scale bars: 5 mm (**A**–**D**), 1 mm (**E**–**H**).

**Figure 5:**
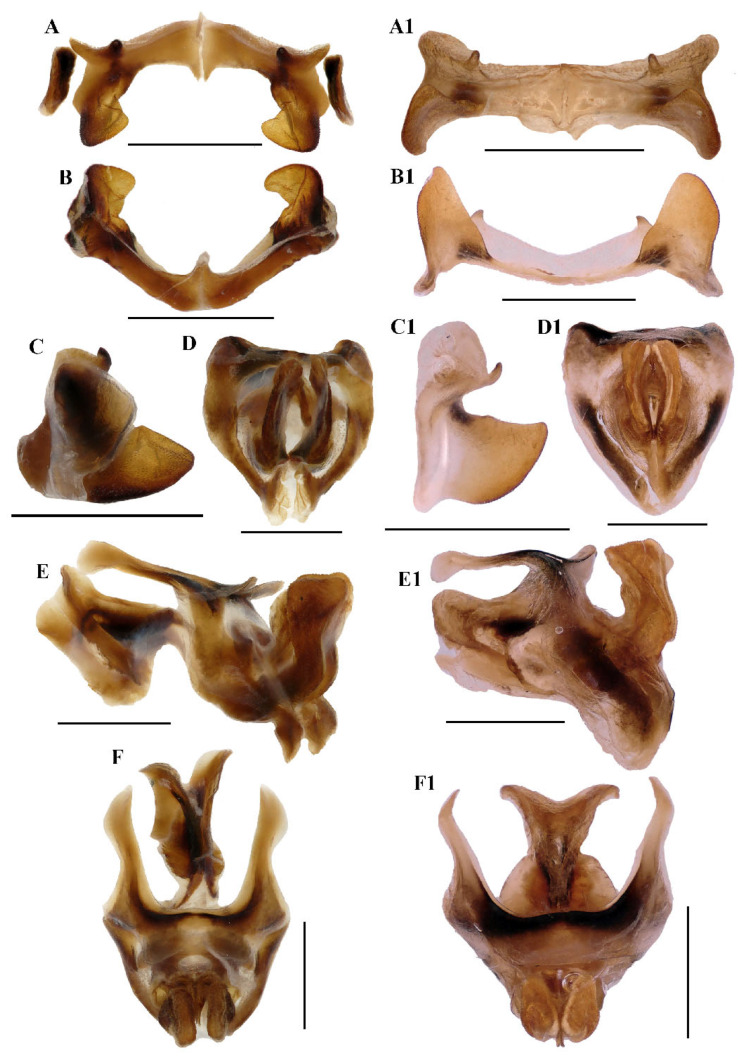
*Caryanda gengmaensis* Mao et Li sp. nov. (**A**–**C**) Epiphallus in dorsal, posterior and lateral views; (**D**–**F**) phallic complex in apical, lateral, and dorsal views. *Caryanda cyanonota* Mao & Li, 2015. (**A1**–**C1**), epiphallus in dorsal, posterior and lateral views; (**D1**–**F1**) phallic complex in apical, lateral and dorsal views. Scale bars: 1 mm (**A**–**F**,**A1**–**F1**).

**Figure 6:**
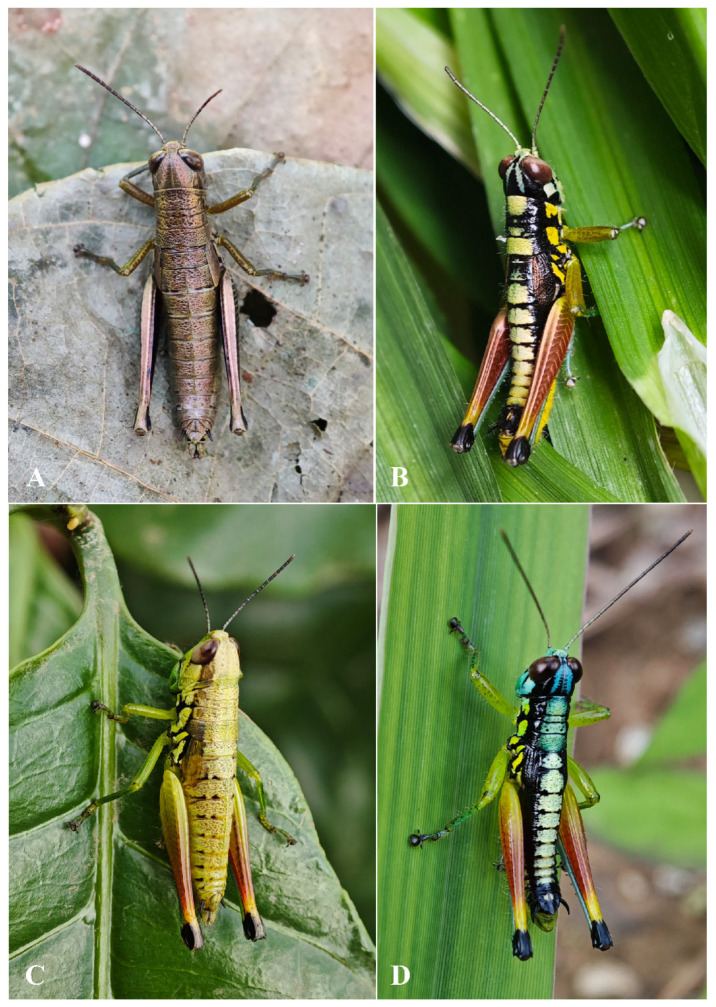
Living photos. (**A**,**B**) *Caryanda gengmaensis* Mao et Li sp. nov. female and male. (**C**,**D**) *Caryanda cyanonota* Mao & Li, 2015. female and male.

Female (Figure 6A) Boby copper with metal color. Hind femora with inner and outer sides brown black; hind tibiae dirty blue. Abdominal terga copper, sterna slightly yellow with a black longitudinal stripe in the middle. Subgenital plate with a pair of ax-shaped black marks. In addition, the color of dried specimens changed from copper color to brown.

**Measurements (mm).** Body length: male 16.50–19.00, female 20.50–23.00; pronotum length: male 3.10–3.80, female 6.20–6.50; tegmen length: male 2.70–3.00, female 2.90–3.60; hind femur length: male 9.40–10.40, female 12.30–12.90.

#### 3.3.2. *Caryanda cyanonota* Mao & Li, 2015 (Figure 4, Figure 5 and Figure 6)

Chinese common name: 青脊卵翅蝗

*Caryanda cyanonota* Mao & Li, 2015: 451, Figures 3, 4 and 11–19 [48].

**Material examined.** 17 males and 8 females (including holotype and paratypes), CHINA: Yunnan, Ximeng, 22°38′ N, 99°35′ E; alt. 1118 m, 1 November 2012, leg. Benyong Mao and Miao Li, deposited in BMDU.

**Notes.** The external morphological features and male genitalia of this species were described by the original author and shown in line drawing, and are illustrated in color here.

#### 3.3.3. *Caryanda analbomaculata* Mao et Li sp. nov. (Figure 7, Figure 9 and Figure 10)

Chinese common name: 拟白斑卵翅蝗

**Type material.** Holotype: male, CHINA: Gengma County, Yunnan Province, 23°38′ N, 99°23′ E, alt. 2046 m, 7 January 2025, leg. Fangting Li, Xun Wang and Honglei Yu. Paratypes: 7 males, 6 females, same data as holotype; 1 male, 2 females, CHINA: Gengma County, Yunnan Province, 29 November 2024, leg. Jianyu Chen and Yongmei Zhao. All type specimens are deposited in BMDU.

**Diagnosis.** This new species is similar to *C. albomaculata* Mao, Ren & Ou, 2007, but differs from the latter in: (1) male 10th abdominal tergite having small furculae on posterior margin (without furculae in *C. albomaculata*, although presence of little vestige of furculae in later specimens); (2) male cerci with apex obviously decurved, obtuse, whereas the apex is weakly decurved and sharper in *C. albomaculata*; (3) the outer lophi of epiphallus with apical inner corner being roundedly acute-angled, whereas the corner nearly rectangular in *C. albomaculata*; (4) the apical valves of penis with broader oval apex in posterior view, and the apex narrower in the latter; (5) the posterior margin of female subgenital plate shallowly concaved in middle and with two obtuse processes on both sides (the posterior margin deeply concaved in middle with two large triangular processes on both sides in the latter; (6) the light marks on male body back being greenish yellow instead of yellowish white in *C. albomaculata*.

**Etymology.** The specific epithet refers to this species being similar to *Caryanda albomaculata* Mao, Ren & Ou, 2007.

**Description.** Body small, stouter in female.

**Figure 7:**
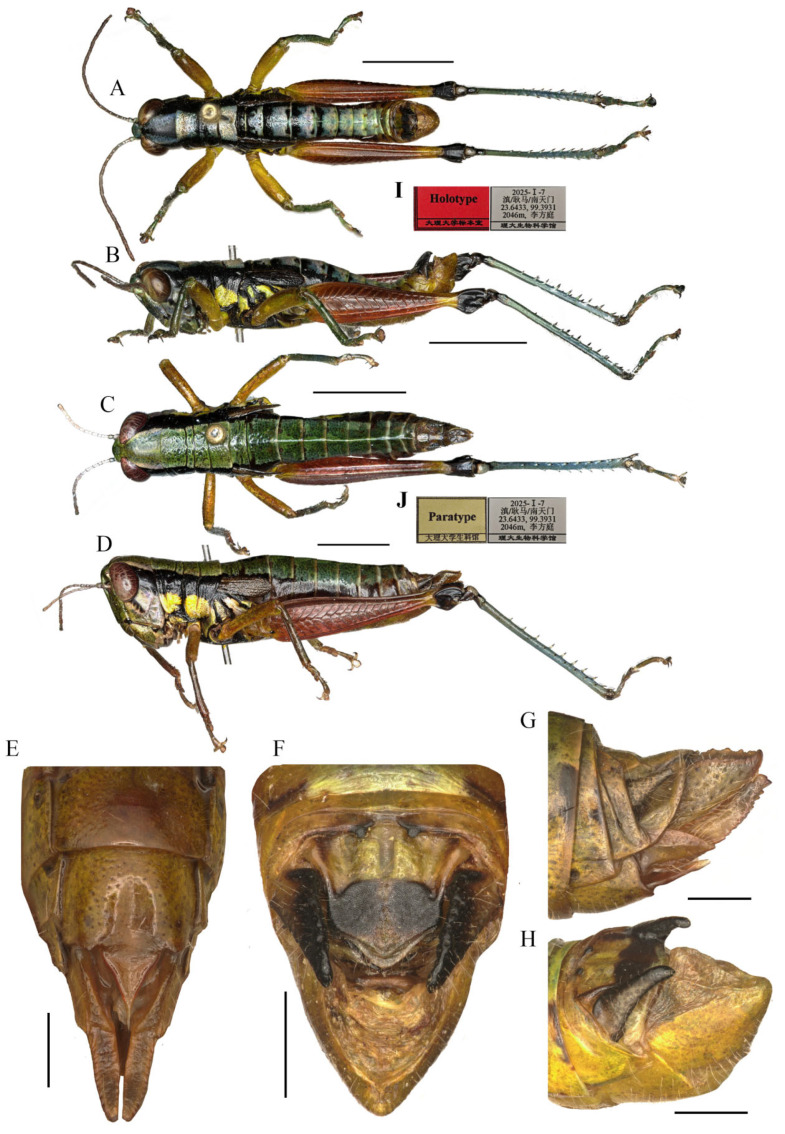
Type specimen picture of *Caryanda analbomaculata* Mao et Li, sp. nov. (**A**,**B**) Male habitus, dorsal and lateral views; (**C**,**D**) female habitus, dorsal and lateral views; (**E**) female terminalia in ventral view; (**F**) male terminalia in dorsal view; (**G**,**H**) female and male terminaliae in lateral view; (**I**,**J**) holotype and paratype labels, respectively. Scale bars: 5 mm (**A**–**D**), 1 mm (**E**–**H**).

**Head** (Figure 7A–D). Head shorter than pronotum; fastigium broad, nearly flat in dorsal view, weakly prominent in lateral view, width in front of eyes about 1.80–2.60 (2.10, mean, n = 5, male) (the same below) or 2.20–3.00 (2.60, female) times larger than length, interocular distance 1.00–1.10 (1.05, male) or 1.10–1.30 (1.20, female) times width of frontal ridge between antennae. Face oblique in profile, surface weakly punctured; frontal ridge laterally straight with longitudinal sulcus in whole length; lateral margins raised, nearly parallel but a little outspread near median ocellus. Lateral facial keels thick and straight. Antennae filiform, retrad reaching the base of hind femora (male) or posterior margin of pronotum (female), median 7–9 segments about 1.80–2.50 (2.10, male) or 1.80–2.30 (1.90, female) times as long as wide. Eyes oval, longitudinal diameter about 1.30–1.50 (1.60, male) or 1.65–1.60 (1.50, female) times as long as horizontal diameter, and about 2.10–2.60 (2.20, male) or 1.90–2.10 (2.00, female) times as long as subocular furrow.

**Figure 8:**
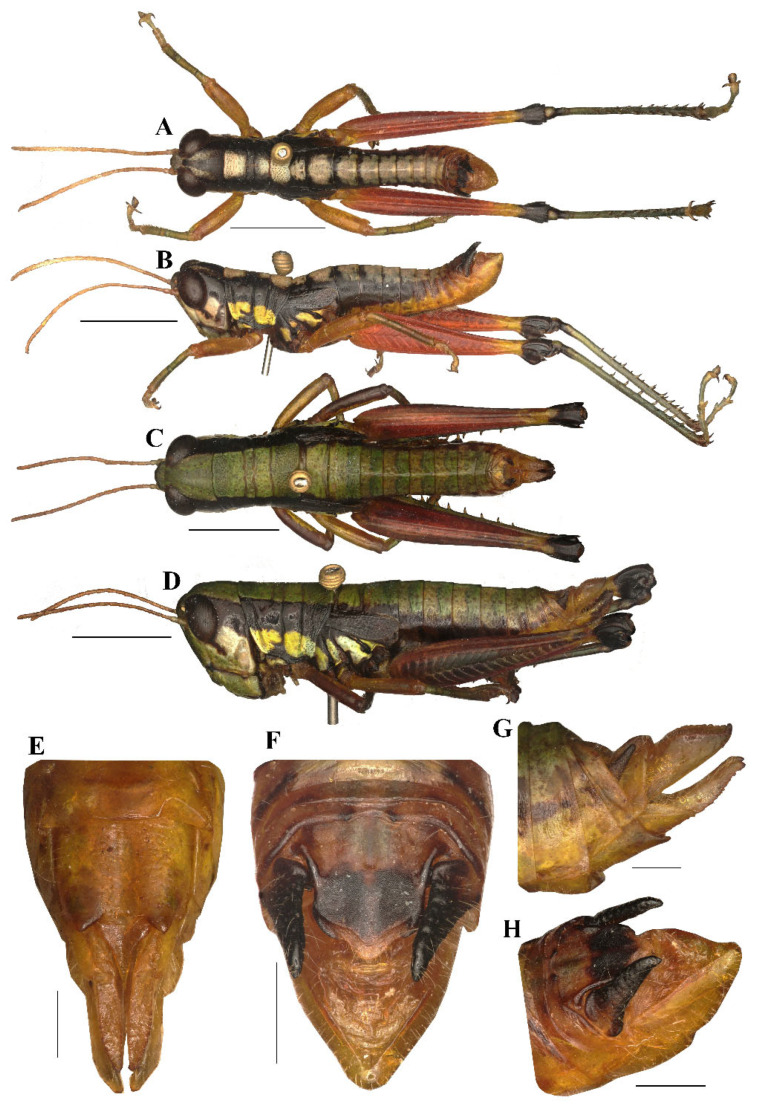
*Caryanda albomaculata* Mao, Ren & Ou, 2007. (**A**,**B**) Male habitus, dorsal and lateral views; (**C**,**D**) female habitus, dorsal and lateral views; (**E**) female terminalia in ventral view; (**F**) male terminalia in dorsal view; (**G**,**H**) female and male terminaliae in lateral view. Scale bars: 5 mm (**A**–**D**), 1 mm (**E**–**H**).

**Figure 9:**
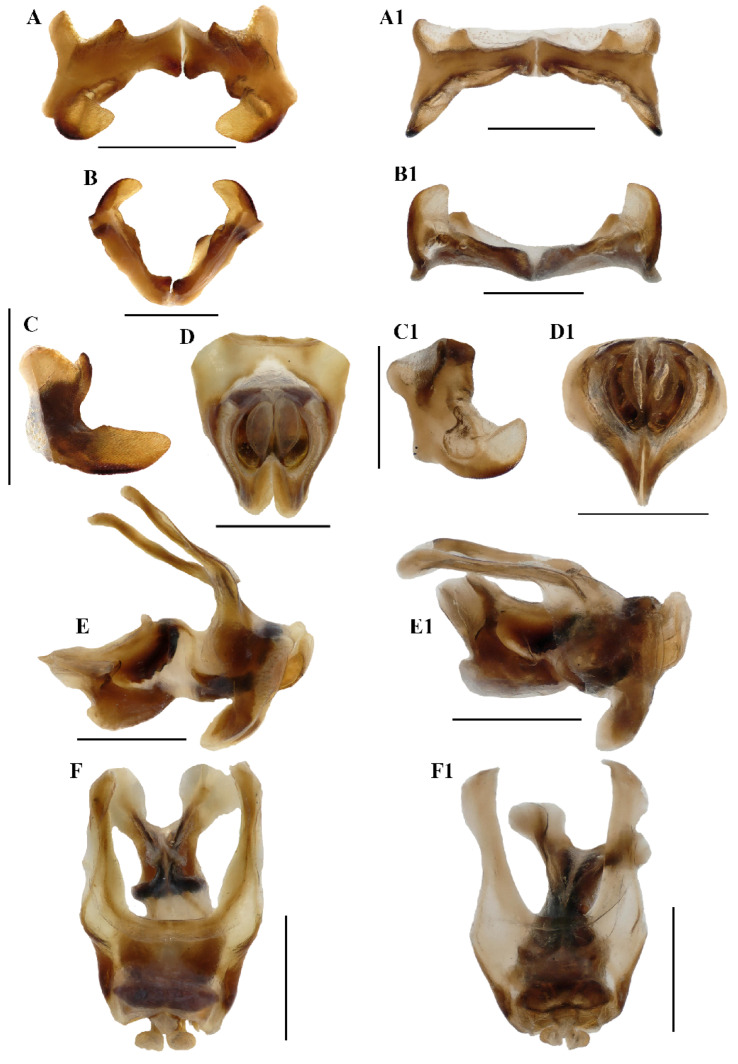
*Caryanda analbomaculata* Mao et Li, sp. nov. (**A**–**C**) Epiphallus in dorsal, posterior and lateral views; (**D**–**F**) phallic complex in apical, lateral, and dorsal views. *Caryanda albomaculata* Mao, Ren & Ou, 2007. (**A1**–**C1**), epiphallus in dorsal, posterior and lateral views; (**D1**–**F1**) phallic complex in apical, lateral, and dorsal views. Scale bars: 1 mm (**A**–**F**,**A1**–**F1**).

**Figure 10:**
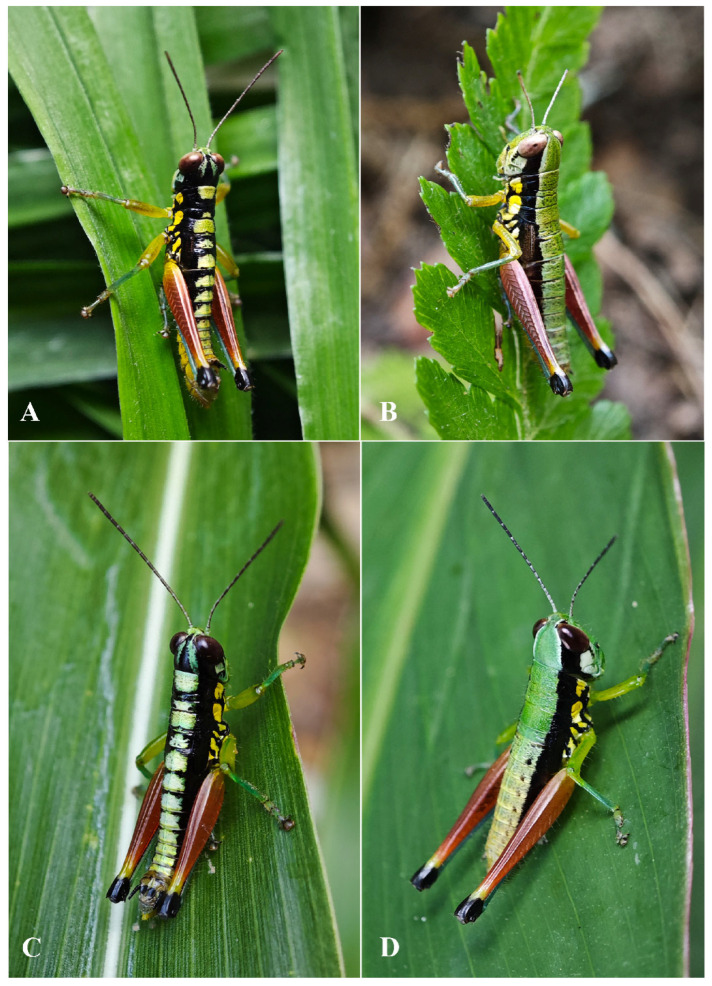
Living photos. (**A**,**B**) *Caryanda analbomaculata* Mao et Li, sp. nov. male and female. (**C**,**D**) *Caryanda albomaculata* Mao, Ren & Ou, 2007 male and female.

**Thorax** (Figure 7A–D). Pronotum nearly cylindrical, slightly contractive in middle, anterior margin nearly straight, posterior margin with a shallow breach; median carina indistinct, lateral carinae absent; three transverse sulci distinct, and have two obvious black spots in middle of pronotum (male); prozona 2.26–2.50 (2.36, on the average, n = 5, male) or 2.20–2.70 (2.60, on the average, n = 5, female) times as long as metazoan, lateral lobe with posterior margin distinctly concave, posteroventral corner obtusely angular. Prosternal spine long conical, straight, apex acute. Mesosternal interspace about 1.60–2.00 (1.87, male) or 1.33–1.50 (1.60 female) times longer than minimum width; mesosteral lobes nearly quadrate, 1.00–1.25 (1.10, male) or 1.10–1.30 (1.20, female) times wider than long; metasternal lobes contiguous (male) or separate (female). Tegmina narrow scale-like, length 2.00–3.00 (2.60, male) or 2.30–2.50 (2.65, female) times larger than maximum width, reaching at or just surpassing beyond posterior margin of 1st abdominal tergite in both sexes (Figure 7B,D). Hind femur with upper carina smooth, terminating in an acute angle; apex of lower knee lobes spinous. Hind tibia with apical half nearly cylindrical, with 7 external and 9 internal spines on dorsal side; external apical spine present (Figure 7A–D). Abdomen with median keel. Tympana opening distinct, oval.

**Abdomen.** Male genitalia (Figure 7F,H). Tenth abdominal tergite widely divided in middle, with small furculae (Figure 7F). Epiproct narrow pentagonal, length at base larger than width, basal half with broad median longitudinal sulcus; lateral margins raised in basal third, weakly contracted in median third; posterior margin triangular with two spine-like processes in lateral sides (Figure 7F). Cerci triangular, surpassing apex of epiproct, pointing post-dorsad, compressed laterally, apex obviously decurved, obtuse. Subgenital plate long conical, apex round (Figure 7H). Epiphallus with outer lophi near parallelogram, apical inner corner roundedly acute-angled, inner lophi small and rounded; ancorae triangular, pointing dorso-apically; bridge divided in middle (Figure 9A–C). Phallic complex (Figure 9D–F): apical valves of penis and cingular valves obviously extended out of hind margin of cingular rami; apical valves of penis upcurved in lateral view, in posterior view apex strongly expanded nearly oval with inner edge retrad rolled; cingular valves upcurved in lateral view, fused apically.

Female genitalia (Figure 7E,G). Epiproct nearly triangular, with a transverse crease in the middle and a middle longitudinal sulcus in the basal half. Cerci conical, apex blunt, not reaching apex of epiproct (Figure 7G). Subgenital plate near quadrate, median area concaved, posterior margin shallowly concaved in middle and with two obtuse processes on both sides (Figure 7E). Valves of ovipositor with dentes along margins.

**Coloration** (Figure 10A,B). The following notes according to fresh specimens. Head with back yellow green, with (male) or without (female) black triangular mark. Antennae brown (male) light red (female). Eyes brown. Postocular bands black, continued to dorsal area of lateral lobes of pronotum, tegmina and 8th abdominal tergite. Pronotum with disk black (male) or green (female), with (male) or without (female) three yellowish green maculations; lateral lobes with two yellow spots, inferior margins black. Tegmina black. Fore and mid legs with femora yellowish green, tibiae blue green. Hind femora dull red, with a yellow ring before knee; knee black; hind tibiae blue. Mesothorax and metathorax black (male) or green (female) with (male) or without (female) two yellowish green maculations on disk, and, respectively, with a yellow spot on episterna and epimera. Abdominal tergites black (male) or green (female) with (male) or without (female) 8 yellowish green maculations on back; abdominal sternites and terminalia yellow (male) or green (female). Cerci and apical half of supra-anal plate black in male. Color of dried specimens is similar to that of fresh ones except light spots discoloring to grayish green on back of male body.

**Measurements (mm).** Body length: male 16.50–18.00, female 19.00–21.30; pronotum length: male 3.10–3.70, female 3.80–6.30; tegmen length: male 2.20–3.00, female 2.50–2.80; hind femur length: male 9.70–11.00, female 10.30–11.50.

#### 3.3.4. *Caryanda albomaculata* Mao, Ren & Ou, 2007 (Figure 8, Figure 9 and Figure 10)

Chinese common name: 白斑卵翅蝗

*Caryanda albomaculata* Mao, Ren & Ou, 2007: 55, Figures 14–26, 30 and 31 [49].

**Material examined.** A total of 12 males and 14 females (including holotype and paratypes), CHINA: Yunnan, Puer, 22°34′ N, 101°11′ E; alt. 1700 m, 28 July 2007, leg. Jishan Xu and Benyong Mao, deposited in BMDU.

**Notes.** The external morphological features and male genitalia of this species were described by the original author and shown in line drawing, and are illustrated in color here.

## 4. Discussion

Species of *Caryanda* are a kind of small-sized flightless grasshoppers with residual wings and weak spreading capability. They have limited distributions in the mountain areas of southern and southwestern Yunnan, where several large rivers, deeply incised valleys, and inter-mountain farmland ecologically isolate mountain habitats. This great habitat heterogeneity is closely related to the uplift of the Qinghai–Tibet Plateau since the Late Pliocene and has also been deeply influenced by human activities recently. Geographic and ecological isolation blocked the gene flow between populations in different habitats, thereby promoting species differentiation that has evolved over tens of millions of years into the current pattern of diversity. Some studies have shown that the divergence time of Acrididae in Yunnan is about 51.94 million years ago (Mya), and the divergence time of *Caryanda* is even later, about 11.68 Mya [50]. There may not be precise limits between incipient taxa due to the complex populations resulting from their geological and evolutionary histories [51]. In this case, mitochondrial genome sequence divergence is often used in Acrididae taxonomy to identify species, especially closely related species [52].

We employed complete mitochondrial genomes to conduct a phylogenetic analysis of four *Caryanda* species bearing pale spots on their backs, alongside three additional species within the same genus. Intergroup genetic distances among the seven *Caryanda* species were calculated using the Kimura 2-parameter (K2P) model within the MEGA 11 software. Results indicate that both Bayesian and maximum likelihood phylogenetic analyses support the monophyly of the two new species. Inter-species genetic distances fluctuate between 2% and 9%, with the two new species exhibiting distances of 2% and 3%, respectively, from their closest relatives. In insect DNA barcoding studies, a genetic divergence of approximately 2–3% (K2P distance) in the mitochondrial COI gene is frequently employed as an empirical reference value for preliminary species differentiation [53,54]. But, the designation of some percentage or degree of divergence as a point below which individuals should be considered conspecific is unrealistic; speciation is not tidy [8]. In Hawlitschek’s research, it is also reasonable for intraspecific p-distances to exceed 2% between populations of the same species at opposite ends of the Palearctic region’s distribution [55]. The range of intraspecific variation differs significantly across different taxonomic groups [51,55,56,57,58]. The two new species proposed by this institute precisely meet the empirical threshold criteria for species definition. Therefore, while the results of genetic distance provide some support for the proposal of these two new species, they cannot serve as an absolute standard for species delimitation [22].

Based on morphological character identification, the differences in external morphological features and genitalia between the two new species and their allied species are stably present. In addition, morphometric results indicate statistically significant differences in seven morphometric measurements between male and female individuals of the two species (*C. analbomaculata* Mao et Li sp. nov., *C. albomaculata* Mao, Ren & Ou, 2007) with a genetic distance of 2% (Table 2 and Table 3). Phylogenetic analyses based on mitochondrial genomic data are consistent with morphological findings. Furthermore, the fact that the two new species have higher inhabiting altitudes (both exceeding 2000 m) and delayed eclosion peaks (appearing around January) than their relatives shows they have different ecological requirements. The combined effect of this altitude gradient and delayed eclosion peaks emergence periods is likely to induce reproductive isolation between the two populations, both spatially and temporally, accompanied by ecological niche differentiation. This process may consequently drive their genetic and phenotypic divergence. Therefore, by integrating results from distinct methodologies, we conclude that the two new species proposed herein are valid. The two new species identified in this study exhibit minimal genetic differentiation from their closest relatives (K2P distances of 2% and 3%, respectively), suggesting that they may represent recently diverged sister species. This hypothesis is supported by morphological and ecological data: Both species exhibit morphologically stable distinguishing characteristics and demonstrate clear ecological niche differentiation (such as habitat elevation and phenological phase shifts). Although these differences are significant, they remain within the range commonly observed among closely related species.

In conclusion, by integrating multi-dimensional evidence encompassing molecular, morphological, and ecological aspects, we confirm the newly described species possesses independent taxonomic status, with its species validity supported by multiple lines of evidence. Two new species within the genus *Caryanda*, originating from Yunnan, are described. This study emphasizes the necessity of integrating multidisciplinary data to define and recognize species, which not only reveals a considerable *Caryanda* species richness in montane Yunnan, but also indicates the influence of the dynamic geological and climatic history of the region in shaping its current diversity.

## Figures and Tables

**Table 1:** Sample information for genomic phylogeny research in this study.

Number	Species and Specimen ID	Habitat Information	Data Sources
1	*C. gengmaensis* Mao et Li sp. nov., 01	Yunnan: Gengma, 23°38′ N, 99°23′ E, alt. 2066 m, 7 January 2025	this study sequencing
2	*C. gengmaensis* Mao et Li sp. nov., 02		this study sequencing
3	*C. analbomaculata* Mao et Li sp. nov., 01	Yunnan: Gengma, 23°38′ N, 99°23′ E, alt. 2046 m, 7 January 2025	this study sequencing
4	*C. analbomaculata* Mao et Li sp. nov., 02		this study sequencing
5	*C*. *cyanonota* Mao & Li, 2015, 01	Yunnan: Ximeng, 22°38′ N, 99°35′ E; alt. 1118 m, 1 November 2012	this study sequencing
6	*C*. *cyanonota* Mao & Li, 2015, 02		this study sequencing
7	*C. albomaculata* Mao, Ren & Ou, 2007, 01	Yunnan: Puer, 22°34′ N, 101°11′ E; alt. 1700 m, 28 July 2007	this study sequencing
8	*C. albomaculata* Mao, Ren & Ou, 2007, 02		this study sequencing
9	*C. albomaculata* Mao, Ren & Ou, 2007, 03		this study sequencing
10	*C. azurea colourfula* Mao, Ren & Ou, 2011, 01	Yunnan: Yuanjiang, 23°06′ N, 102°45′ E; alt. 1731 m, 29 July 2009.	this study sequencing
11	*C. azurea colourfula* Mao, Ren & Ou, 2011, 02		this study sequencing
12	*C*. *neoelegaus* Otte, 1995	/	NC 036750
13	*C*. *xinpingensis* Mao, 2017	/	KU375571
14	*Alulatettix yunnanensis* Liang, 1993	/	NC 018542
15	*Euparatettix bimaculatus* Zheng, 1993	/	NC 046541.1

**Table 2:** *C. analbomaculata* Mao et Li sp. nov. and *C. albomaculata* statistical significance test for quantitative trait measurements and correlation ratios in males.

Sample	BL	PNL	HFL	HFW	EL	EW	CL	CW	HFL/ HFW	EL/ EW	CL/ CW
Can_m1	18	6.1	9.2	2.2	2.9	2.8	2.5	1.2	4.18	1.04	2.08
Can_m2	17.7	6.2	9.7	2.1	2.9	2.6	2.7	1.3	4.62	1.12	2.08
Can_m3	18.4	6.1	9.7	2.1	2.8	3	2.6	1.3	4.62	0.93	2.00
Can_m4	17.5	6.2	9.7	2	2.6	2.7	2.6	1.2	4.85	0.96	2.17
Can_m5	17.6	6.2	9.9	2.1	2.7	2.9	2.7	1.2	4.71	0.93	2.25
Can_m6	18.7	6.7	9.8	2.2	3	3	2.9	1.2	4.45	1.00	2.42
Can_m7	18.5	6.6	9.9	2.1	2.8	3	2.6	1.2	4.71	0.93	2.17
Can_m8	18.6	6.4	10.1	2.1	2.7	3	2.6	1.2	4.81	0.90	2.17
Ca_m1	19	6.1	10.1	2.1	2.9	3	2.8	1.3	4.81	0.97	2.15
Ca_m2	19	6.9	10.4	2.1	2.9	3.2	3	1.5	4.95	0.91	2.00
Ca_m3	19.9	7	10.3	2.1	3.1	3.2	1.9	1.4	4.90	0.97	1.36
Ca_m4	18.2	6.5	10	2	2.8	3.3	2.8	1.3	5.00	0.85	2.15
Ca_m5	19.3	6.7	10.5	2.2	2.8	3.2	2.9	1.2	4.77	0.88	2.42
Ca_m6	19	6.8	10.2	2.1	2.7	2.8	2.7	1.4	4.86	0.96	1.93
Ca_m7	19.1	6.5	10.4	2.1	2.8	3	2.8	1.5	4.95	0.93	1.87
Ca_m8	18.9	6.4	10.2	2.1	2.9	3.2	2.7	1.4	4.86	0.91	1.93
Ca_m9	18.6	6.9	10.6	2.2	2.8	2.9	2.8	1.2	4.82	0.97	2.33
Ca_m10	18.5	6.5	10.3	2.1	2.7	3.2	2.8	1.4	4.90	0.84	2.00
Ca_m11	18	6.2	9.3	2	2.7	2.8	2.6	1.3	4.65	0.96	2.00
Ca_m12	18.2	6.5	9.6	2.1	2.5	2.7	2.9	1.6	4.57	0.93	1.81
Ca_m13	19.2	6.7	10.2	2	2.8	2.9	3	1.2	5.10	0.97	2.50
Ca_m14	19	7	11	2.1	3	2.7	3.1	1.4	5.24	1.11	2.21
Ca_m15	19	6.7	9.7	2.9	2.8	3.1	2.6	1.4	3.34	0.90	1.86
W value	16.5	23.5	21	65	56.5	37	28	18	22.5	76.5	84
*p* value	0.0052	0.0190	0.0126	0.7399	0.8415	0.1397	0.0385	0.0051	0.0168	0.2974	0.1276

Note: The sample codes from “Can_m1” to “Can_m8” represent the measured male individuals of *C. analbomaculata* Mao et Li sp. nov., and the “Ca_m1” to “Ca_m15” represent the measured male individuals of *C. albomaculata*. The acronyms for measurements are as follows: HFL/HFW–Ratio of HFL to HFW, EL/EW–Ratio of EL to EW, CL/CW–Ratio of CL to CW.

**Table 3:** *C. analbomaculata* Mao et Li sp. nov. and *C. albomaculata* statistical significance test for quantitative trait measurements and correlation ratios in females.

Sample	BL	PNL	HFL	HFW	FSPL	FSPW	HFL/HFW	FSPL/FSPW
Can_f1	19.2	7.3	11	2.3	2.7	2.8	4.78	0.96
Can_f2	20.6	8.4	11.4	2.5	2.8	2.8	4.56	1.00
Can_f3	21	8	11.3	2.4	3	2.8	4.71	1.07
Can_f4	20.8	7.9	11.1	2.3	2.8	2.8	4.83	1.00
Can_f5	20	7.4	/	/	3	2.7	/	1.11
Ca_f1	21.5	8	11.5	2.4	2.8	3.6	4.79	0.78
Ca_f2	23	8.4	12.3	2.6	2.5	3.1	4.73	0.81
Ca_f3	21.7	8.3	12.3	2.6	3	3.5	4.73	0.86
Ca_f4	22.5	8.2	12.2	2.6	2.8	3.7	4.69	0.76
Ca_f5	20	8.4	12.2	2.6	3	3.5	4.69	0.86
Ca_f6	21.2	8.2	12	2.5	3.3	3.2	4.80	1.03
Ca_f7	22.5	9.1	13.1	2.6	2.8	3.8	5.04	0.74
Ca_f8	21.8	7.9	12.2	2.5	3	3.5	4.88	0.86
Ca_f9	23	8.2	12.5	2.6	2.8	3.2	4.81	0.88
Ca_f10	22.8	8.5	12.2	2.7	3.2	3.2	4.52	1.00
W value	3.5	9	0	2.5	20	0	17	45
*p* value	0.0099	0.0554	0.0052	0.0117	0.5617	0.0023	0.7231	0.0162

Note: The sample codes from “Can_f1” to “Can_f8” represent the measured female individuals of *C. analbomaculata* Mao et Li sp. nov., and the “Ca_f1” to “Ca_f15” represent the measured female individuals of *C. albomaculata*. The acronyms for measurements are as follows: FSPL/FSPW–Ratio of FSPL to FSPW, others same as above.

## Data Availability

The original contributions presented in this study are included in the article. Further inquiries can be directed to the corresponding authors.
